# Foraging ecology of three sympatric ungulate species – Behavioural and resource maps indicate differences between chamois, ibex and red deer

**DOI:** 10.1186/s40462-015-0033-x

**Published:** 2015-03-14

**Authors:** Anna K Schweiger, Martin Schütz, Pia Anderwald, Michael E Schaepman, Mathias Kneubühler, Rudolf Haller, Anita C Risch

**Affiliations:** Remote Sensing Laboratories, Department of Geography, University of Zurich, Winterthurerstrasse 190, 8057 Zürich, Switzerland; Research Unit Community Ecology, Swiss Federal Institute for Forest, Snow and Landscape Research WSL, Zürcherstrasse 111, 8903 Birmensdorf, Switzerland; Department of Research and Geoinformation, Swiss National Park, Chastè Planta-Wildenberg, 7530 Zernez, Switzerland

**Keywords:** Biomass, Forage, Imaging spectroscopy, Movement, Nitrogen, Remote sensing, Resource selection, T-LoCoH, Utilisation distribution, Vegetation

## Abstract

**Background:**

The spatial distribution of forage resources is a major driver of animal movement patterns. Understanding where animals forage is important for the conservation of multi-species communities, since interspecific competition can emerge if different species use the same depletable resources. However, determining forage resources in a spatially continuous fashion in alpine grasslands at high spatial resolution was challenging up to now, because terrain heterogeneity causes vegetation characteristics to vary at small spatial scales, and methods for detection of behavioural phases in animal movement patterns were not widely available. We delineated areas coupled to the foraging behaviour of three sympatric ungulate species (chamois, ibex, red deer) using Time Local Convex Hull (T-LoCoH), a non-parametric utilisation distribution method incorporating spatial and temporal autocorrelation structure of GPS data. We used resource maps of plant biomass and plant nitrogen content derived from high-resolution airborne imaging spectroscopy data, and multinomial logistic regression to compare the foraging areas of the three ungulate species.

**Results:**

We found significant differences in plant biomass and plant nitrogen content between the core foraging areas of chamois, ibex and red deer. Core foraging areas of chamois were characterised by low plant biomass and low to medium plant nitrogen content. Core foraging areas of ibex were, in contrast, characterised by high plant nitrogen content, but varied in plant biomass, and core foraging areas of red deer had high plant biomass, but varied in plant nitrogen content.

**Conclusions:**

Previous studies carried out in the same study area found no difference in forage consumed by chamois, ibex and red deer. Methodologically, those studies were based on micro-histological analysis of plant fragments identifying them to plant family or functional type level. However, vegetation properties such as productivity (biomass) or plant nutrient content can vary within vegetation communities, especially in highly heterogeneous landscapes. Thus, the combination of high spatial resolution resource maps with a utilisation distribution method allowing to generate behavioural maps (T-LoCoH) provides new insights into the foraging ecology of the three sympatric species, important for their conservation and to monitor expected future changes.

**Electronic supplementary material:**

The online version of this article (doi:10.1186/s40462-015-0033-x) contains supplementary material, which is available to authorized users.

## Background

How ecologically similar species coexist in a shared habitat is a fundamental question in ecology [1]. Resource ecology provides the basis for understanding multi-species assemblages as it deals with plant-nutrient relationships, interactions between consumers and resources and interactions among consumers [2]. Foraging is the central process in resource ecology as it leads to growth, survival and reproduction of the animal and thus, ultimately influences its fitness [2]. Ungulates forage selectively [3] and are not only influenced by vegetation and landscape structure, but are themselves major drivers of landscape heterogeneity [4-8]. Additionally, ungulates affect the abundance and population dynamics of other species, ranging from herbivores [9] to soil decomposers [10] which in turn feed back to vegetation composition and structure. These traits make the spatial distribution and foraging ecology of ungulates an important issue in wildlife management, nature protection and landscape conservation [11].

The diversity of ungulate communities is often explained by differences in their dietary niches [12]. Most studies have investigated forage selection based on plant family or functional type (e.g. graminoids, forbs, shrubs) level and have used either direct observations [13,14], fence experiments [13,15] or micro-histological analysis of faecal pellets [16-20]. Thus, ungulates are traditionally categorised according to their feeding types as grazers, mixed feeders or browsers (concentrate selectors) [3,12]. Previous studies defined chamois (*Rupicapra rupicapra* L.), ibex (*Capra ibex* L.) and red deer (*Cervus elaphus* L.) as mixed feeders, with chamois being closer to browsers, ibex closer to grazers and red deer in between [12,13,21]. Such similarity of dietary niches would imply high potential for competition among the three species, especially when population numbers are high. In the Trupchun valley of the Swiss National Park (SNP) population sizes of sympatric chamois, ibex and red deer are amongst the highest in central Europe. Previous studies in this area investigated the forage composition of the three species using micro-histological analysis of faecal pellets and found no significant differences in the proportions of grasses, sedges, forbs and woody species consumed during spring and summer [22-24].

However, large variations in forage composition at the plant species and plant family level were not only reported between, but also within ungulate species (e.g. for deer species see [3]) suggesting flexibility in their dietary choices. Additionally, plant species within a vegetation type can strongly differ in growth form and nutritious value resulting from small scale heterogeneity of microclimate and soil, which is especially pronounced in alpine landscapes [25]. Thus, vegetation type classifications might conceal the heterogeneity of forage resources [26]. High-resolution remote sensing has demonstrated the potential to detect environmental heterogeneity [27,28] at a spatial scale fine enough to be relevant for foraging animals [3,26,29]. Advanced observational approaches such as imaging spectroscopy (IS [30,31]) make it possible to detect changes in plant biochemical and biophysical composition [28,32,33], and plant species distribution [34]. Plant biomass and plant nitrogen (N) content are vegetation characteristics important for forage resource selection in ungulates [35-39] and have already been mapped successfully using IS in heterogeneous grassland ecosystems [35,40-42].

The home range (HR) of an animal is the area traversed by the individual during its normal activities of food gathering, mating and caring for offspring [43]. Advances in global positioning system (GPS) technology have made it possible to collect large amounts of location data [44,45] and several HR estimators (polygon methods) - from minimum convex polygons (MCP) to alpha hulls [46], kernel density estimators (KDE) and local convex hulls (LoCoH) [47] - have been proposed.

Traditional HR estimators have been criticised for treating locations as spatially and temporally independent, an assumption that can only be fulfilled when data are collected either at random [48] or at time intervals long enough to allow an animal to move to any place within its HR [49]. However, it has been argued that efforts to handle spatial autocorrelation, which can be an intrinsic data attribute [50], have drawn attention away from more important questions in HR analysis [51]. Instead of removing spatial autocorrelation, which has been shown to be of limited relevance for HR estimators (e.g. KDE) it can be used as a source of biological information and therefore be incorporated in models of animal movement and space use [52].

Similarly, polygon methods have been criticised for giving only limited information about the species’ biology when focussing on the perimeter (size and shape) of an HR. Thus, additional insights into the species’ biology might be gained (i.e. what the animal did and where) by using spatial and temporal autocorrelation to delineate areas coupled to the animal’s behaviour [48]. During the last decade, models of space use incorporating temporal autocorrelation of GPS data became more widely available, including (dynamic) Brownian bridge movement models (BBMM) [53,54], Levy flight movement models [55], movement based kernel density estimators (MKDE) [56] and time geography methods [57,58]. Similarly, behavioural models (models of time-use) that take advantage of the temporal autocorrelation of GPS data, such as cognitive models [59] or state-space models [60] were developed.

One of the few methods that take both spatial and temporal autocorrelation of GPS data into account is the Time Local Convex Hull approach (T-LoCoH) [61]. T-LoCoH introduces time-scaled distance (TSD), which measures the distance between two points in both space (using the x/y coordinates) and time (using timestamps) allowing to calculate time-use metrics, such as directionality of movement, duration of stay or revisitation rate of a specific area [61]. These metrics can be used to generate behavioural maps serving as proxies to delineate migration corridors, resting or foraging areas. Core foraging areas have been defined as regions within an HR that are most heavily used for foraging [62] and have been approximated by taking the 10% to 50% isopleths of an animal’s utility distribution (UD; see [63,64]). T-LoCoH’s ability to detect behavioural phases using the temporal and spatial autocorrelation structure of GPS data [61] fills an important gap in HR [50] analyses.

Ungulates are mobile, have accurate spatial memory [11], spend most of their time feeding [65] and allocate their time according to the resources available [8,66]. Thus, areas frequently revisited by the animals can be expected to contain important forage resources. Thanks to the SNP’s long term monitoring and behavioural studies [67,68] we know that ungulates in the SNP follow daily movement patterns between foraging sites and are active during most time of the day, likely caused by limited disturbances (strong protection status, absence of predators). Red deer are known to follow a bimodal diurnal rhythm (peaks around sunrise and sunset) in areas strongly influenced by human activities [69], while behaving polyphasal (several activity peaks during day and night) when human disturbance is low [70].

The goal of our study was to investigate the potential of combining high-resolution remote sensing data with a HR estimator incorporating the behavioural information contained in GPS data for studying a classical issue in resource ecology, resource partitioning between sympatric species. We used GPS data and T-LoCoH's revisitation index to delineate the core foraging areas of the three ungulate species, chamois, ibex and red deer, co-occurring at high population densities in the Trupchun valley of the SNP and airborne IS data to map plant biomass (forage quantity) and plant N content (forage quality) at 2 m × 2 m spatial resolution. We compared vegetation characteristics in the core foraging areas of the three ungulate species with multinomial logistic regression and related our results to previous studies examining their diet composition.

## Methods

### Study area

Our study was carried out in the Trupchun valley (46.6° N, 10.08° E) of the SNP, encompassing approximately 22 km^2^ close to the Italian border. Elevation in the Trupchun valley ranges from 1775 to 3145 meter above sea level (m a.s.l.), the average annual temperature in the SNP is 0.9 ± 0.5°C (mean ± SD) and the mean precipitation is 754 ± 164 mm (2004–2013, recorded at the park’s weather station at 1977 m a.s.l.) [71]. The plant’ growing season lasts from mid May until mid September. The Trupchun valley is known for its high numbers of co-occurring ungulates; population estimates between 9–10 chamois/km^2^, 10–11 ibex/km^2^ and 25–31 red deer/km^2^ were reported in 2010–2013 [64].

### Vegetation data

We collected vegetation data allowing validation of IS data based models in 51 (2010, 2011) to 100 plots (2012, 2013), covering the entire range of exposition, altitude, productivity and plant species composition in the Trupchun valley. These plots were 6 m × 6 m in size, homogenous in vegetation cover and species composition and were grouped into five clusters to enable harvesting within a short time frame after the APEX overflight (approximately four hours; see [41] for details). Georeferencing of the plots was performed using a high-precision GNSS (Global Navigation Satellite System) receiver (Leica 1200+, Leica Geosystems, Heerbrugg, Switzerland) with measurement accuracy < 1 cm. On the day of overflight, 1 m^2^ of vegetation was clipped in the centre of each plot and immediately sealed into plastic bags. We weighed the samples the same day to determine fresh weight of plant biomass. Then the samples were dried at 65°C and milled to pass a 0.5 mm screen (Pulverisette 16, Fritsch, Idar-Oberstein, Germany). One third of the vegetation samples were chemically analysed for total plant N and plant NDF (neutral detergent fibre) content using standard laboratory methods (TruSpec CN analyser Leco Corp., St Joseph, MI, USA; Fibre Analyser 200, Ankom Technology, NY, USA). Plant NDF content is a widely used indicator of forage quality and important for ruminal function in ungulates [72]. The reflectance spectra of the vegetation samples were measured using a laboratory near-infrared reflectance spectrometer (NIRS; Multi-purpose near-infrared reflectance spectrometer (NIR-MPA), Bruker Optics, Switzerland) and chemically analysed samples were subsequently used to calibrate models for predicting plant N content and plant NDF content of all vegetation samples. NIRS models achieved predictive accuracies of R^2^ = 0.93 for plant N and R^2^ = 0.81 for plant NDF content. Since we found strong correlation between plant N content and plant NDF content (R^2^ = − 0.61, p < 0.001), we excluded plant NDF content from further analysis. When comparing the vegetation characteristics in the plots sampled in all four years (n = 25), using Wilcoxon rank sum tests for pairwise comparisons, no significant differences regarding plant biomass (all p ≥ 0.19) and plant N content (all p ≥ 0.35) were found. This allowed us to combine GPS data of the animals collected in different years (but always within 43 days of the APEX flight) with the corresponding IS data sets.

### Imaging spectroscopy data

Imaging spectroscopy (IS) data were collected on June 24, 2010, June 26, 2011, June 29, 2012 and July 12, 2013 using the airborne imaging spectrometer APEX [31,73], mounted on a propeller aircraft (Dornier DO-228) operated by the German Aerospace Centre (DLR). APEX covers the wavelength region between 380 nm and 2500 nm in 334 reconfigurable spectral bands. After removing noisy bands, 285 (2010), 301 (2011), 299 (2012) and 284 (2013) spectral bands remained for analysis. Ground pixel size depended on flight altitude, but was resampled to 2 m x 2 m. APEX IS data were geometrically and atmospherically corrected using the software packages PARGE [74] and ATCOR-4 [75], based on the atmospheric radiative transfer code MODTRAN-5. Geometric mis-registration of the orthorectified data was evaluated using ground-based differential global positioning system (DGPS) measurements and was found to be less than one pixel (± 2 m) in flat terrain [76] and up to two pixels (± 4 m) on steep slopes (A. Damm, personal communication). Generally, IS data collected at different times are not comparable due to differences in sun angle and atmospheric conditions resulting in varying surface anisotropy. Therefore, we used APEX IS data and ground reference vegetation data to model forage quantity and quality for each year separately. Since reference plots measured 6 m × 6 m and APEX pixel size was 2 m × 2 m, a 3 × 3 pixel aggregation scheme was defined to extract the reflectance values from the IS data per plot.

We calculated simple ratios indices (SRI = band i/band j) for all possible band combinations based on the average reflectance of the 9 aggregated pixels per plot and determined the correlation between plant biomass (g.m^−2^) and plant N (%) content and the SRI using Pearson’s correlation coefficient (R^2^). Next, we used the SRI’s with the 100 highest correlations (according to R^2^) as input to model plant biomass and plant N content with linear, exponential and second order polynomial functions and validated the models using leave-one-out cross validation (see also [41]). We selected the best model according to Akaike’s Information Criterion (AIC) and evaluated model fit with Theil’s uncertainty coefficient (Theil’s U). Compared to Pearson correlation, Theil’s U has the advantage of taking deviations of the slope from its ideal value of 1 and deviations of the intercept from its ideal value of 0 into account [77]. Theil’s U normalizes the sum of the squared prediction errors between observed and predicted values to a value between zero and one, with zero indicating perfect agreement [77]. Generally, values of Theil’s U < 0.2 indicate high, values between 0.2 and 0.4 moderately high predictive power. Due to their frequent use, we also added R^2^ values in our text and graphs. Additionally, we determined predictive accuracy by calculating the root mean squared error of prediction (RMSE) and the proportion of samples predicted within less than 20% RMSE. Finally, we applied the best models to predict and map plant biomass and plant N content in all 2 m × 2 m raster cells of the grasslands in the Trupchun valley. Since our models were designed to predict plant biomass and plant N content only in grasslands, we used linear spectral unmixing (LSU) and applied a 50% threshold to exclude areas dominated by forest, rock, snow or water from mapping [78]. IS data were prepared using ENVI (version 4.7, Exelis Visual Information Solutions, Boulder, CO, US). All analyses were conducted in R [79]. For the map layout we used ArcGIS (version 10.1, Environmental Systems Research Institute, Redlands, CA, US).

### GPS data collection

To match the temporal scale of IS data collection we used GPS data from five chamois (two in 2011, one in 2012, two in 2013), seven ibex (four in 2010, three in 2013) and two red deer (both in 2013) recorded within three weeks before and three weeks after the APEX IS flights (total of 43 days per year). All animals were caught and handled by SNP rangers experienced in the procedures and regularly supervised by a veterinarian. Chamois and female ibex were caught in box traps and marked without narcosis. Red deer and male ibex were darted and injected with 1 ml to 3 ml Hellabrunner Mischung (125 mg Xylazin + 100 mg Ketamin per ml), dependent on body weight. The animals were released within 30 minutes after an injection of 1 ml to 3 ml Antipamezol, an antagonistic drug. The animals were equipped with GPS PLUS collars (Vectronic Aerospace GmbH, Berlin, Germany). The fix rate was set to either four or two hours, but was resampled to the common interval of four hours during data preparation. We performed a GPS accuracy test, placing two collars at six georeferenced (Leica GNSS 1200+, see above) locations, two in the main valley, two in the forest and two in the grasslands of the Trupchun valley. We placed the collars around wooden frames with heights ranging from 120 cm to 140 cm and rotated the collars between the locations on a weekly basis. The location error of the collars was 11.3 ± 4.7 m (mean ± SD; [= SQRT ((SD (x-coordinate)^2) + (SD (y-coordinate)^2))] ). If the animal was captured or recaptured during the 43 day time window around the APEX flights, the first and last days of data collection were excluded from analysis. GPS data were screened for unrealistic movement following the method of Bjørneraas et al. [80], with limiting parameters set to α = 1.5 km/h and cos θ = − 0.97 (velocity and turning angle defining erroneous turnarounds, i.e. spikes in the data), μ = 50 km (possible distance travelled within 20 h) and Δ = 200 km (distance impossible to travel within 20 h; for details see [80]).

### Behavioural maps

We used the T-LoCoH package in R [81], a non-parametric UD method to construct behavioural maps [61] that serve as proxy to delineate the core foraging areas of the three ungulate species. T-LoCoH models space use by constructing local MCP’s or hulls around each data point, which are then sorted and progressively merged to form isopleths. Sorting of the hulls can be based on different time-use metrics that serve as proxies for the animals’ behaviour, such as duration of stay, directionality of movement and revisitation rates. The time-stamp of each location is incorporated in both, the selection of nearest neighbours for local hull construction and the sorting of the hulls. For hull construction, two points have to be close in time and in space to be considered nearest neighbours. T-LoCoH introduces a distance function that transforms a unit of time into a unit of distance, called time-scaled distance (TSD). The time and space components of TSD are weighted by setting parameter “s”. To make comparisons between the animals possible, we used the same process for all individuals and species and set “s” to a consistent proportion of 60% time selected hulls [61,82].

The number of nearest neighbours can be defined by selecting the “k” closest points in space and time (“k-method”), the points within a defined time-scaled radius “r” (“r-method”) or by identifying the nearest neighbours up to a cumulative distance “a” in space and time (“a-method”). We decided to use the “a-method” as this method is better suited for studies where both, high and low point densities of GPS locations can be expected [47]. As before, we used the same process to define parameter “a” for all individuals of all species. We set parameter “a” to a cumulative distance that stabilised the isopleths’ edge to area ratio [61,82] before creating a jump in the isopleths’ area, thus balancing type I (including area that is not used) and type II errors (omitting area that is used) [83]. Since absolute values for the optimal “a” across all individuals of a specific ungulate species were very close, it was reasonable to use the same value for “a” for all three species.

While there are guidelines available for selecting the weight placed on the time-component (“s”-value) and the threshold for nearest neighbour selection (“a”-value) [61], the parameters for hull sorting and the isopleths’ threshold have to be based on the aim of the study and the knowledge of the animals’ ecology. In our study area, the three ungulate species show distinct diurnal movement patterns. They are known to regularly return to the same areas for foraging, ruminating and resting and have multiple activity peaks per day [67,68].We therefore calculated the revisitation rate for each hull based on an inter-visit gap (IVG, time to pass for an observation to count as a separate visit) of 12 hours, sorted the hulls according to the mean number of separate visits normalised (NNSV) and merged them until 30% of all points were included (creating the 30% isopleths). While it is likely crucial to limit type II errors (omitting areas important for a species) in conservation projects (e.g. the delineation of protected areas), studies of animal behaviour profit from limiting type I errors (including area that is not used by the species) to detect the patterns of interest. Thus we decided to choose a tight threshold, i.e. the 30% isopleths, to delineate the animals’ core foraging areas.

### Species comparison

We fitted multinomial logistic regression models using the three ungulate species as the response and plant biomass and plant N content in the animals’ core foraging areas as predictor variables. We rescaled plant biomass to a level similar to plant N content by dividing all biomass values by 100 (BiomRS = Biomass/100). As candidate models we chose i) the two models containing only one predictor variable (plant biomass or plant N content), ii) the main effects model containing both terms (plant biomass and plant N content), iii) the model including both terms plus their interaction and iv) the intercept-only model. We selected the best model based on differences of AIC (Δ AIC) and confirmed our selection using the likelihood ratio test. To evaluate model fit we calculated the Hosmer-Lemeshow goodness-of-fit statistic and the area under curve (AUC) of the receiver-operating characteristic (ROC) for each of the two logits separately [84]. The ROC is obtained by plotting all sensitivity values (true-positive fraction) on the y-axis against their equivalent 1-specificity values (false-positive fraction) for all thresholds on the x-axis. Thus, this measure of overall accuracy is independent of any threshold [85]. AUC values between 0.7 and 0.8 indicate good, values between 0.8 and 0.9 excellent discriminative ability [86]. We assessed the sensitivity of our results with regard to the size of the animals’ core foraging areas by re-running the analysis after adding and subtracting a 6 m buffer to the core foraging areas, respectively, and tested the hypothesis of equality of the model coefficients. For analyses and graphs we used the packages nnet [87], pROC [88] and effects [89] in R [79].

## Results

**Figure 1:**
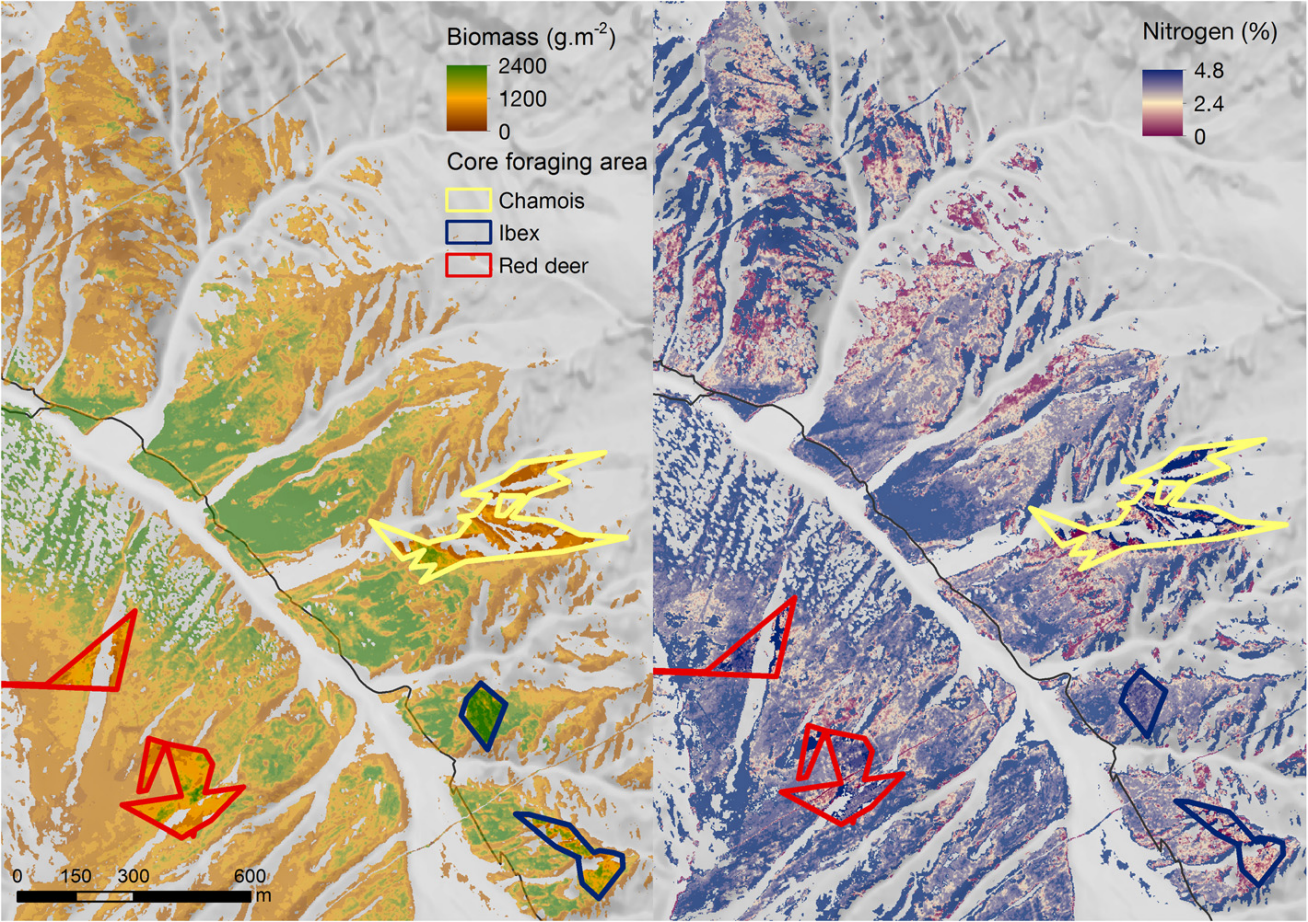
**Examples of core foraging areas of chamois, ibex and red deer, respectively.** Core foraging areas represent the 30% isopleths of T-LoCoH’s revisitation index. The map shows fresh weight of plant biomass (g.m^−2^) (left panel) and plant nitrogen content (%) (right panel). Grey colours represent areas covered by forest, rock, snow or water, identified using linear spectral unmixing (LSU) and subsequently excluded from analysis.

The SRI models of grassland vegetation developed from APEX IS data predicted plant biomass and plant N content with high to moderately high predictive power (Table 1). Generally, the grasslands in the Trupchun valley showed high heterogeneity regarding plant biomass and plant N content (Table 2). The core foraging areas of chamois, ibex and red deer delineated using T-LoCoH's revisitation index (NNSV) were in agreement with the areas where the three ungulate species are frequently observed (SNP, personal communication). Figure 1 shows examples of spatially segregated core foraging areas in the Trupchun valley. We found minor overlaps of the core foraging areas both within and between species, however they occurred at different times of our observation period. The best multinomial logistic regression model included plant biomass, plant N content and their interaction (df = 8, Table 3). When comparing the other candidate models to this model (the interaction model), the main effects model reached a Δ AIC of 38.6 (df = 6), the model including only plant N content a Δ AIC of 668.0 (df = 4), the model including only plant biomass a Δ AIC of 2013.1 (df = 4) and the intercept-only model a Δ AIC of 2538.8 for (df = 2). According to likelihood ratio tests, the interaction model performed clearly better (all p < 0.001) than all other models. Therefore, we chose the interaction model as our best model. The Hosmer-Lemeshow goodness-of-fit statistic for each of the two logits reached a value of p < 0.001 indicating very good model fit. The best model’s AUC was 0.82 for logit 1, indicating excellent ability to discriminate between chamois and ibex coreforaging areas, and 0.75 for logit 2, indicating good ability to discriminate between chamois and red deer coreforaging areas. Sensitivity analysis revealed no significant differences (all p > 0.25) between the best model's coefficients for the two logits, neither after increasing, nor after decreasing the animals’ core foraging areas by a 6 m buffer (see Additional file 1).

**Table 1:** **Imaging spectroscopy models predicting fresh weight of plant biomass and plant nitrogen content**

		**Biomass (g.m** ^**−2**^ **)**	**Nitrogen (%)**
2010	Theil’s U	0.19	0.11
	adj. R^2^	0.65	0.53
	RMSE	174.37	0.53
	< 20 % RMSE (%)	44.19	62.79
2011	Theil’s U	0.15	0.07
	adj. R^2^	0.70	0.43
	RSME	155.71	0.28
	< 20 % RMSE (%)	53.57	88.80
2012	Theil’s U	0.23	0.07
	adj. R^2^	0.49	0.39
	RSME	174.35	0.26
	< 20 % RMSE (%)	36.44	84.48
2013	Theil’s U	0.22	0.08
	adj. R^2^	0.43	0.36
	RSME	241.30	0.27
	< 20 % RMSE (%)	36.93	81.67

Models were generated separately for each year using data from the imaging spectrometer APEX and in situ vegetation data. Model performance is described using Theil’s uncertainty coefficient (Theil’s U), adjusted Pearson’s correlation coefficient (adj. R^2^), root mean squared error of prediction (RMSE) and % of predicted values below 20 % RMSE (< 20 % RMSE (%)).

**Table 2:** **Resources in the Trupchun valley and in core foraging areas of chamois, ibex and red deer**

	**Trupchun**	**Chamois**	**Ibex**	**Red deer**
Mean biomass (g.m^−2^)	295.26	192.28	242.63	276.79
SD biomass (g.m^−2^)	230.10	84.83	119.75	107.15
Biomass min (g.m^−2^)	0.10	32.48	0.10	83.84
Biomass max (g.m^−2^)	2799.10	667.61	680.30	986.63
Mean nitrogen (%)	2.11	1.84	2.37	1.78
SD nitrogen (%)	0.53	0.25	0.64	0.22
Nitrogen min (%)	0.01	1.29	0.97	1.04
Nitrogen max (%)	4.80	2.75	4.75	3.69

Biomass (g.m^−2^) = fresh weight of plant biomass (g.m^−2^), N (%) = plant nitrogen content (%), SD = standard deviation, min = minimum, max = maximum.

**Table 3:** **Best multinomial logistic regression model comparing chamois, ibex and red deer core foraging areas**

	**Variable**	**Coeff**	**SE**	**z**	**p**
Logit 1	Intercept	−8.7218	0.5825	−14.9738	0.0000
Chamois vs. ibex	BiomRS	1.0043	0.2658	3.7786	0.0002
	N	3.6048	0.2898	12.4381	0.0000
	BiomRS:N	−0.1314	0.1369	−0.9602	0.3369
Logit 2					
Chamois vs. deer	Intercept	2.2434	0.6461	3.4722	0.0005
	BiomRS	−0.1548	0.2731	−0.5670	0.5707
	N	−2.4917	0.3538	−7.0432	0.0000
	BiomRS:N	0.5535	0.1471	3.7640	0.0002

Logit 1 represents the logistic link function for chamois vs. ibex core foraging areas, logit 2 the logistic link function for chamois vs. red deer core foraging areas. Coefficients of the parameters (Coeff) for plant biomass rescaled (BiomRS = fresh weight of plant biomass/100 (g.m^−2^)), plant nitrogen content (N (%)) and their interaction (BiomRS:N), standard errors (SE), Wald Z-statistic values (z) and corresponding p-values (p) are indicated.

**Figure 2:**
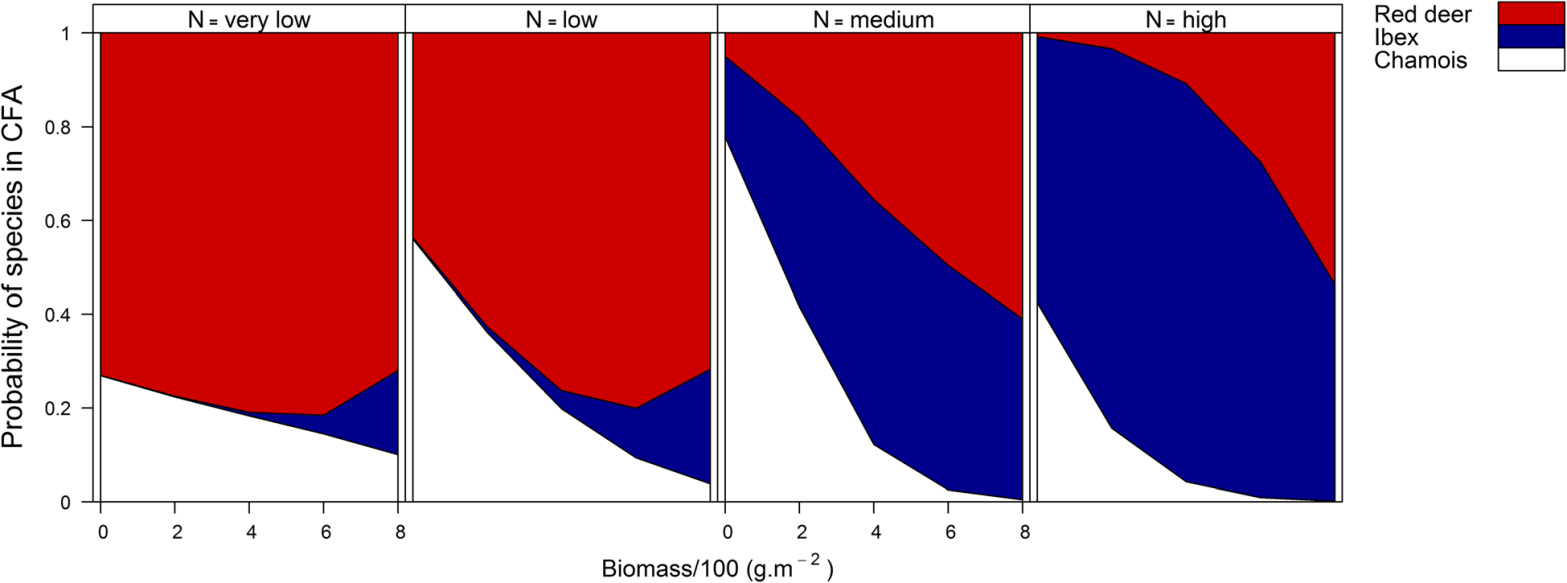
**Probabilities for de facto use of core foraging areas by red deer, ibex and chamois.** Predicted probabilities for chamois, ibex and red deer using core foraging areas (CFA) depending on plant biomass (Biomass/100 (g.m^−2^), x-axis) at increasing levels of plant nitrogen content (very low (<1%), low (< 2%), medium (< 2.5%), high (≥ 2.5%)) displayed in the panels from left to right. Probabilities were generated from the model in Table 3.

**Figure 3:**
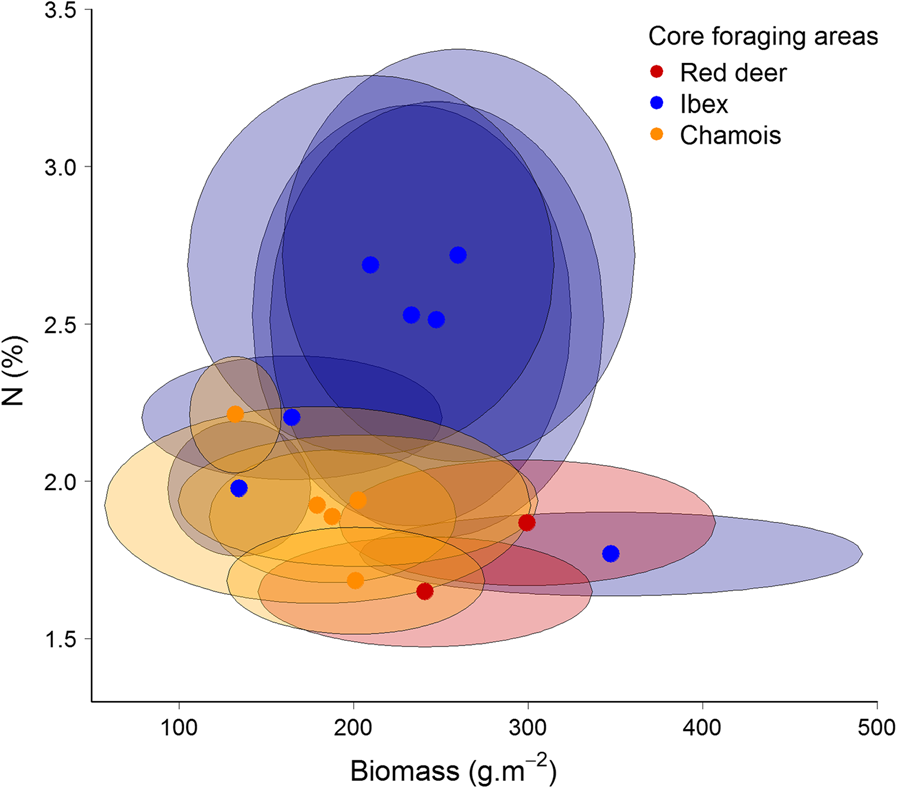
**Core foraging areas of red deer, ibex and chamois.** Core foraging areas of red deer, ibex and chamois regarding plant biomass and plant nitrogen (N) content. Dots represent mean values and the axes of the ellipses standard deviations (SD) in either direction.

**Figure 4:**
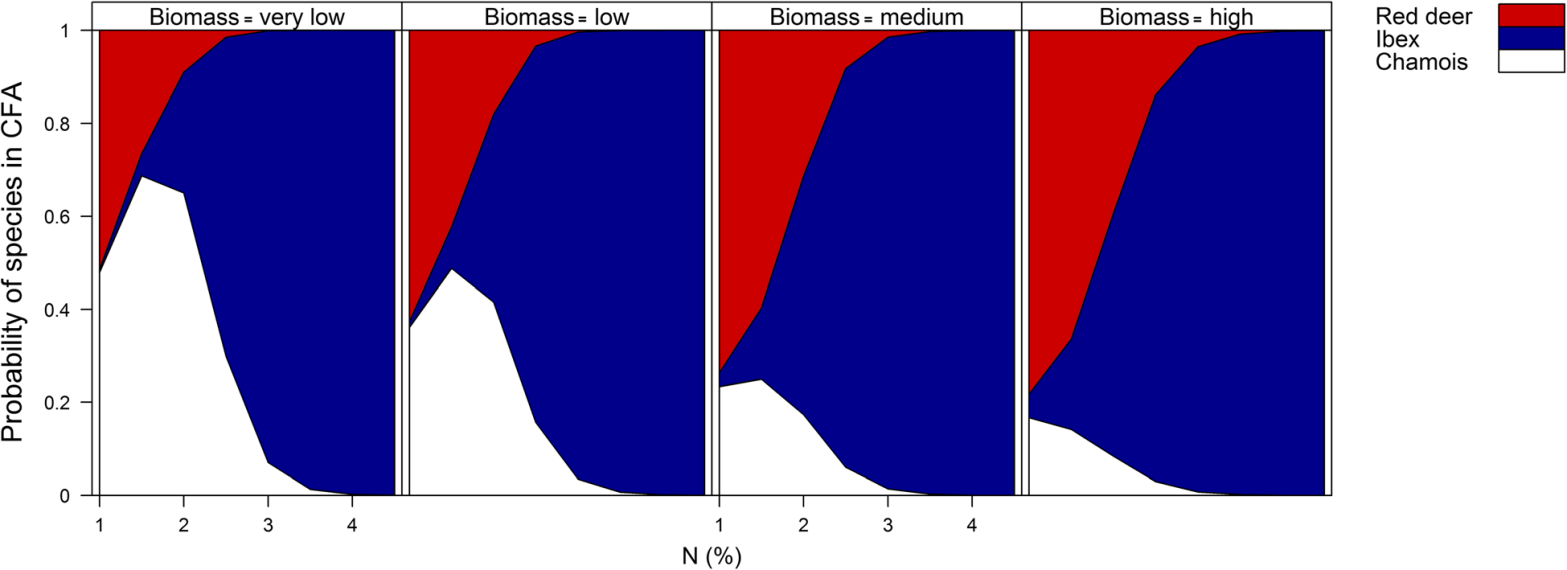
**Probabilities for de facto use of core foraging areas by red deer, ibex and chamois.** Predicted probabilities for chamois, ibex and red deer using core foraging areas (CFA) depending on plant nitrogen content (x-axis) at increasing levels of plant biomass (very low (< 200 g.m^−2^), low (< 350 g.m^−2^), medium (< 450 g.m^−2^), high (≥ 450 g.m^−2^)) displayed in the panels from left to right. Probabilities were generated from the model in Table 3.

The core foraging areas of chamois were characterised by generally low plant biomass (< 200 g.m^−2^ fresh weight, Figures 2 and 3), and a low but slightly increased level of plant N content (around 2%, unimodal relationship, Figures 3 and 4). In contrast, vegetation in the core foraging areas of ibex was characterised by high plant N content, but variable plant biomass (Figure 2, Figure 4), while vegetation in the core foraging areas of red deer was characterised by high levels of plant biomass, but variable plant N content (Figure 2). Ibex showed a tendency to use areas with the highest plant biomass and plant N content (Figure 3). However, the core foraging areas with the highest plant biomass and highest plant N content had an almost 50:50 modelled chance of being used by either ibex or red deer (Figure 2).

## Discussion

Previous studies conducted in the Trupchun valley found no difference in spring and summer forage composition between chamois, ibex and red deer when using micro-histological analysis of plant fragments in faecal pellets [22-24]. Likewise, a large overlap in the diet of chamois and red deer was found when they co-occurred with roe deer (*Capreolus capreolus* L.) in Southern Germany [90], with mouflon (*Ovis ammon musimon* Pallas) in the Western Alps [17] and with re-introduced red deer in the Italian Apennine [20]. While overlap in resource use (of both habitat and forage) is a prerequisite for competition [91], it could also be a sign of coexistence between species with no need of specialisation or segregation [92]. Similarly, low overlap in diet and high specialisation may point towards species living in coexistence [1], but could also be an effect of active competition, with the species trying to relieve competitive pressure [92]. This makes coexistence and competition extremely difficult to demonstrate in the field and without experimental manipulation [91,93,94], and conclusion have to be drawn with care.

The studies mentioned above used micro-histological analyses of faecal pellets and thus identified forage remains at the level of plant functional groups or plant families. However, the ratios of the two main forage components, graminoids and forbs, varied considerably within the ungulate species studied, which suggests that the animals have some flexibility in their dietary choices. Moreover, ungulates are assumed to partition forage resources at levels below the scale of vegetation types [3], and several studies confirm that plant biomass, plant nutrient and mineral content are major drivers for the spatial distribution and forage resource selection in ungulates [35-39]. Therefore, studies investigating forage resource selection in multi-species ungulate communities profit from including forage quantity and quality in their analyses, especially in areas where these vegetation characteristics are expected to vary.

Finding core foraging areas of chamois predominantly where plant biomass was low, red deer core foraging areas where plant biomass was high and ibex in between is in line with traditional feeding type definitions [12]. Regarding body size, chamois as the smallest of our three study species (body weight: 30 – 50 kg) is more limited in terms of forage intake than ibex (body weight: 40 – 150 kg) and red deer (body weight: 60 – 200 kg). However, the differences of plant N content in the core foraging areas of the three species, with chamois foraging in areas with low, ibex in areas with high and red deer in areas with variable plant N content warrant some explanation. Chamois have smaller and less complex rumens resulting in shorter retention time of digesta. This makes them less able to digest fibre, which could indicate that chamois depend on forage with higher plant N content [12]. However, it was found that by comparison to red deer, chamois foraged on lower quality vegetation consisting predominantly of graminoids in areas where food supply was limited [95]. Additionally, chamois have been found to select high quality forage in high quality habitats, while foraging in a more generalist pattern in low quality habitats [14]. Indeed, as mixed feeders [12] chamois can be expected to show high plasticity in forage selection, which was supported by our results.

In contrast to chamois, vegetation in the core foraging areas of ibex was characterised by high plant N content and variable plant biomass. Generally, we expected ibex to forage in rocky terrain with little, but nutrient-rich vegetation. While our results suggested that ibex did indeed forage in areas where plant biomass was low but of high nutritious value (high plant N content), we also found ibex core foraging areas in the highest quality meadows of the Trupchun valley where both plant biomass and plant N content were high. Generally, terrain roughness and slope create a template of risk [96,97], in which herbivores have to trade off between resource acquisition (e.g. foraging in high quality habitats, finding mates) and predator avoidance [98,99]. Ibex are very good climbers that find protection from predators and the possibility to overview large areas in predominantly rocky terrain with steep slopes. Within the SNP predators are absent, hunting is prohibited and visitors are obliged to stay on the marked paths. Thus, ibex might have abandoned part of their anti-predator behaviour in favour of maximising forage resource acquisition. Visual observations (SNP, unpublished observations) confirm that the rather flat, high quality meadows are regularly visited by ibex, where they forage together with red deer and occasionally also chamois.

The core foraging areas of red deer were always located on the rather flat meadows where the animals are expected to be able to cover their forage intake needs as plant biomass is high. Red deer inhabit predominantly open, flat terrain, have good running skills and thus withdraw themselves from predator attacks by using areas with high lateral cover, such as areas with forest or tall-growing shrubs. Similar to ibex, red deer might have abandoned part of their anti-predator behaviour as they can be observed grazing, resting and ruminating on the alpine meadows of the Trupchun valley during daytime [67,68]. However, besides the absence of predators and limited disturbance, finding ungulates foraging in open areas with higher plant biomass could also be an effect of high population densities [100]. When forage availability in habitats with more protection declines with increasing population numbers, the animals might be forced to use more open terrain for foraging. To assess whether the animals choose the high-quality meadows in the Trupchun valley voluntarily (in order to maximise resource acquisition) or if they are forced to use these areas (due to high population numbers) would require a comparison of core foraging areas at variable population densities or in the presence of predators. Wolf, lynx and bear are expected to return to the SNP in the future [101], which could have profound impacts on the abundance, population dynamics and spatial distribution of ungulates [102]. Thus, our results provide an important basis against which to assess future changes.

Due to the fixed dates of the APEX IS data acquisition, the results of our study represent the animals’ behaviour during a specific time, i.e. during early summer. However, forage quantity and quality are expected to influence ungulate movement patterns in our study area in particular during this time of the year (peak of the plant growing season), when females have to nourish their offspring, and all individuals have to build up winter reserves. Naturally, the quantity and quality of forage resources will change during the course of the year and therefore also the ungulates’ habitat use patterns can be expected to change [103,104]. The habitat use patterns of ungulates are apart from vegetation quantity and quality, also influenced by physical landscape characteristics, such as elevation, aspect and slope, as they can facilitate e.g. effective temperature regulation [104-106], ease of movement and anti-predator behaviour [98,99]. However, vegetation composition and thus the quantity and quality of forage resources depend on microclimate and soil, which are also influenced by elevation, aspect and slope. Disentangling the effects of “pure” physical landscape characteristics and “derived” vegetation properties would be challenging but provide important insights into trade-off mechanisms in habitat choice.

The distribution of vegetation quantity and quality influences the space use of herbivores on several spatial and temporal scales [104,107]. Large ungulates show HR establishment at the regional or landscape scale, they choose suitable feeding areas and vegetation communities at the local scale, select vegetation communities of favourable quantity and quality at the patch scale and certain plant species or plant parts at the bite scale [41,107]. APEX data gathered in this study represents vegetation quantity and quality at the patch scale of 2 m x 2 m. It is therefore possible, that some of the ungulate species, especially the smaller chamois, feed more selectively within these patches [3,11,108]. However, visually observing and exactly locating the animals, sampling browsed plants and determining their nutrient content is difficult in an area where access is limited due to challenging terrain and the high protection status. As advances in GPS and remote sensing technology continue, spatially accurate, high-temporal resolution GPS data (e.g. at minute intervals) that allow following the exact movement paths of animals will become more widely available. Combined with temporally flexible, very high spatial resolution remote sensing instruments such as unmanned aerial vehicles (UAV’s, drones; [109]), this would provide opportunities to investigate forage quantity and quality at the individual plant level. Further differentiation might be achieved by not only assessing commonly observable vegetation traits by remote sensing [110], but also by adding advanced retrievals of pigment composition [111].

## Conclusions

We detected significant differences in plant biomass and plant N content in the core foraging areas of sympatric chamois, ibex and red deer when combining resource maps developed from airborne imaging spectroscopy data with behavioural maps developed using the T-LoCoH algorithm: T-LoCoH enables to detect behavioural phases in GPS data by making use of their temporal and spatial autocorrelation. The combination of behavioural and resource maps proved to be valuable for studying a classical issue in resource ecology, resource partitioning between sympatric species. For the future, we expect that the development of remote sensing instruments with increased spatial resolution and temporal flexibility together with highly accurate and short interval GPS systems will continue to deepen our understanding of the foraging ecology of multi-species communities.

## Additional file

Additional file 1:
**Multinomial logistic regression results after adding and subtracting a 6 m buffer around the animals’ core foraging areas (CFA), respectively.** Logit 1 represents the logistic link function for chamois vs. ibex core foraging areas, logit 2 the logistic link function for chamois vs. red deer core foraging areas. Coefficients of the parameters (Coeff) for plant biomass rescaled (BiomRS = fresh weight of plant biomass/100 (g.m^-2^)), plant nitrogen content (N (%)) and their interaction (BiomRS:N), standard errors (SE), Wald Z-statistic values (z), corresponding p-values (p) and the p-values for the hypothesis test for equality of the model coefficients (p (Anova)) are indicated.
