# Supplementary material for: Foraging ecology of three sympatric ungulate species – Behavioural and resource maps indicate differences between chamois, ibex and red deer
Source: Mov Ecol. 2015 Mar 14;3:6. doi: 10.1186/s40462-015-0033-x (PMC4722786; doi:10.1186/s40462-015-0033-x)
Supplement: Additional file 1: — Multinomial logistic regression results after adding and subtracting a 6 m buffer around the animals’ core foraging areas (CFA), respectively. Logit 1 represents the logistic link function for chamois vs. ibex core foraging areas, logit 2 the logistic link function for chamois vs. red deer core foraging areas. Coefficients of the parameters (Coeff) for plant biomass rescaled (BiomRS = fresh weight of plant biomass/100 (g.m-2)), plant nitrogen content (N (%)) and their interaction (BiomRS:N), standard errors (SE), Wald Z-statistic values (z), corresponding p-values (p) and the p-values for the hypothesis test for equality of the model coefficients (p (Anova)) are indicated. [file 40462_2015_33_MOESM1_ESM.docx]

| Original CFA | Variable | Coeff | SE | z | p |  |
| --- | --- | --- | --- | --- | --- | --- |
| Logit 1 | BiomRS | 1.0043 | 0.2658 | 3.7786 | 0.0002 |  |
| Chamois vs. Ibex | N | 3.6048 | 0.2898 | 12.4381 | 0.0000 |  |
|  | BiomRS:N | -0.1314 | 0.1369 | -0.9602 | 0.3369 |  |
| Logit 2 | BiomRS | -0.1548 | 0.2731 | -0.5670 | 0.5707 |  |
| Chamois vs. Deer | N | -2.4917 | 0.3538 | -7.0432 | 0.0000 |  |
|  | BiomRS:N | 0.5535 | 0.1471 | 3.7640 | 0.0002 |  |
|  |  |  |  |  |  |  |
| Original CFA plus 6 m buffer | Variable | Coeff | SE | z | p | p (Anova) |
| Logit 1 | BiomRS | 0.6565 | 0.2147 | 3.0580 | 0.0022 | 0.8542 |
| Chamois vs. Ibex | N | 3.0942 | 0.2245 | 13.7804 | 0.0000 | 0.3736 |
|  | BiomRS:N | 0.0416 | 0.1104 | 0.3768 | 0.7063 | 0.4445 |
| Logit 2 | BiomRS | -0.1122 | 0.2240 | -0.5010 | 0.6164 | 0.6459 |
| Chamois vs. Deer | N | -2.8967 | 0.2951 | -9.8173 | 0.0000 | 0.2593 |
|  | BiomRS:N | 0.5692 | 0.1208 | 4.7120 | 0.0000 | 0.6289 |
|  |  |  |  |  |  |  |
| Original CFA minus 6 m buffer | Variable | Coeff | SE | z | p | p (Anova) |
| Logit 1 | BiomRS | 1.2643 | 0.3302 | 3.8285 | 0.0001 | 0.9947 |
| Chamois vs. Ibex | N | 3.9514 | 0.3674 | 10.7537 | 0.0000 | 0.8007 |
|  | BiomRS:N | -0.2640 | 0.1705 | -1.5481 | 0.1216 | 0.4863 |
| Logit 2 | BiomRS | -0.4466 | 0.3502 | -1.2754 | 0.2022 | 0.6022 |
| Chamois vs. Deer | N | -2.4704 | 0.4506 | -5.4824 | 0.0000 | 0.4080 |
|  | BiomRS:N | 0.6697 | 0.1892 | 3.5403 | 0.0004 | 0.6009 |
